# Effective re-induction regimen for children with recurrent medulloblastoma

**DOI:** 10.1093/noajnl/vdae070

**Published:** 2024-05-10

**Authors:** Katrina O’Halloran, Sheetal Phadnis, Gregory K Friedman, Katie Metrock, Tom B Davidson, Nathan J Robison, Benita Tamrazi, Jennifer A Cotter, Girish Dhall, Ashley S Margol

**Affiliations:** Keck School of Medicine of University of Southern California, Los Angeles, California, USA; Department of Pediatrics, Children’s Hospital Los Angeles, Los Angeles, California, USA; Department of Pediatrics, University of Alabama at Birmingham, Birmingham, Alabama, USA; Department of Pediatrics, Children’s of Alabama, Birmingham, Alabama, USA; Department of Pediatrics, University of Alabama at Birmingham, Birmingham, Alabama, USA; Department of Pediatrics, Children’s of Alabama, Birmingham, Alabama, USA; Division of Pediatrics, The University of Texas MD Anderson Cancer Center, Houston, Texas, USA; Department of Pediatrics, University of Alabama at Birmingham, Birmingham, Alabama, USA; Department of Pediatrics, Children’s of Alabama, Birmingham, Alabama, USA; Keck School of Medicine of University of Southern California, Los Angeles, California, USA; Department of Pediatrics, Children’s Hospital Los Angeles, Los Angeles, California, USA; Keck School of Medicine of University of Southern California, Los Angeles, California, USA; Department of Pediatrics, Children’s Hospital Los Angeles, Los Angeles, California, USA; Keck School of Medicine of University of Southern California, Los Angeles, California, USA; Department of Radiology, Children’s Hospital Los Angeles, Los Angeles, California, USA; Keck School of Medicine of University of Southern California, Los Angeles, California, USA; Department of Pathology and Laboratory Medicine, Children's Hospital Los Angeles, Los Angeles, California, USA; Department of Pediatrics, University of Alabama at Birmingham, Birmingham, Alabama, USA; Department of Pediatrics, Children’s of Alabama, Birmingham, Alabama, USA; Keck School of Medicine of University of Southern California, Los Angeles, California, USA; Department of Pediatrics, Children’s Hospital Los Angeles, Los Angeles, California, USA

**Keywords:** medulloblastoma, recurrence, re-induction

## Abstract

**Background:**

There is no standard treatment for the recurrence of medulloblastoma, the most common malignant childhood brain tumor, and prognosis remains dismal. In this study, we introduce a regimen that is well-tolerated and effective at inducing remission.

**Methods:**

The primary objectives of this study were to assess tolerability of the regimen and overall response rate (ORR). A retrospective chart review of patients with recurrent medulloblastoma, treated at two institutions with a re-induction regimen of intravenous irinotecan and cyclophosphamide with oral temozolomide and etoposide, was performed. Demographic, clinicopathologic, toxicity, and response data were collected and analyzed.

**Results:**

Nine patients were identified. Median age was 5.75 years. Therapy was well-tolerated with no therapy-limiting toxicities and no toxic deaths. Successful stem cell collection was achieved in all 5 patients in whom it was attempted. ORR after 2 cycles was 78%. Three patients had a complete response, 4 patients had a partial response, 1 patient had stable disease, and 1 patient had progressive disease. Four patients are alive with no evidence of disease (NED), 2 patients are alive with disease, 2 patients have died of disease, and 1 patient died of toxicity related to additional therapy (NED at time of death).

**Conclusions:**

This regimen is well-tolerated and effective. Tumor response was noted in the majority of cases, allowing patients to proceed to additional treatment with no or minimal disease. Further study of this regimen in a clinical trial setting is an important next step.

## Lay Summary

Medulloblastoma is a common brain cancer in children. There are no standard therapies when the disease comes back after initial treatment. In this study, the authors looked to see how well a new combination chemotherapy consisting of intravenous irinotecan and cyclophosphamide with oral temozolomide and etoposide treated medulloblastoma once the disease came back after initial treatment. To do this, they analyzed the medical records of 9 medulloblastoma patients treated with this regimen across 2 U.S. institutions. They found no unexpected side effects from the intensive chemotherapy treatment. A positive effect was seen in 7 of the 9 patients after two rounds of treatment with tumors shrinking substantially or disappearing completely. All patients were able to go on to receive additional help to control disease.

**Key Points**

A re-induction regimen of intravenous irinotecan and cyclophosphamide with oral temozolomide and etoposide is well tolerated and effective for some patients with recurrent medulloblastoma.

**Importance of the Study**

There is no standard of care for recurrent or progressive medulloblastoma, and prognosis remains dismal. To improve outcomes, it is necessary to identify regimens that are effective in inducing radiographic remission, especially in order to augment therapies that work best in the setting of minimal disease. Toxicity is also a significant concern, given that these patients have already received myelosuppressive therapy. This study highlights a novel and well-tolerated regimen that successfully induces responses in some patients, allowing them to proceed to additional therapies with no or minimal disease burden. It may be considered a therapeutic option for patients with recurrent medulloblastoma and should be considered for incorporation into future clinical trials.

Medulloblastoma is the most common malignant brain tumor in children, requiring intensive multimodal therapy inclusive of aggressive surgical resection followed by craniospinal irradiation (CSI) and chemotherapy. High-dose chemotherapy (HDC) with autologous stem cell rescue (AuSCR) is often employed to avoid CSI in infants and young children due to the known neurocognitive late effects of cranial irradiation in this age group.^1–5^ Despite these therapies, 20-30% of children diagnosed with medulloblastoma will experience tumor recurrence with the majority of those dying from disease.^1,4,6–9^ At the time of medulloblastoma recurrence, the salvage approach depends on which upfront therapy was used, with consideration for the alternative treatment strategy. In this study, we outline a tolerable and effective re-induction regimen for children with recurrent medulloblastoma.

## Medulloblastoma Recurrence After HDC and AuSCR

It is well-described that a proportion of children treated with irradiation-avoiding strategies upfront can be salvaged with full-dose CSI with or without neo-adjuvant, adjuvant, or maintenance chemotherapy.^10–13^ Salvage CSI has been shown to have overall survival (OS) rates of 39% at five years.^14^ A more recent international retrospective study of patients with relapse after CSI-avoiding upfront therapy reported a 5-year post-relapse survival of 42.6% with a majority of patients subsequently receiving salvage CSI.^15^

## Medulloblastoma Recurrence After CSI

In contrast, patients who experience recurrence after upfront treatment with CSI have dismal outcomes. A review of the Canadian experience reported that 31.2% of pediatric patients diagnosed with medulloblastoma had recurrence (85% received CSI during upfront therapy), and OS at 5 years following recurrence was 12.4%.^16^ The Seattle group reported a similar experience, with median survival after disease recurrence of 10.3 months and 3-year overall survival of 18% for patients treated with upfront CSI.^17^ Patients treated with CSI-containing regimens are typically treated at recurrence with either salvage conventional chemotherapy or HDC with AuSCR.^13,18,19^

## Recurrence After CSI—Conventional Chemotherapy Salvage

The Children’s Oncology Group (COG) phase II study of the use of temozolomide in patients with recurrent central nervous system tumors showed activity against medulloblastoma and tumors previously designated as primitive neuroectodermal tumor (PNET) with an overall response rate (ORR) of 16%.^20^ Subsequently, 4 cycles of temozolomide in combination with irinotecan was shown to have an ORR of 32.6%.^21^ The COG study ACNS0821 (NCT01217437) evaluated the addition of bevacizumab and demonstrated that 17.4% of patients had a complete response (CR), and 30.4% had a partial response (PR; ORR 47%) with prolonged OS by 3 months compared to temozolomide and irinotecan without bevacizumab.^22^

## Recurrence After CSI—HDC and AuSCR Salvage

Salvage strategies utilizing HDC and AuSCR have also been described in the treatment of recurrent medulloblastoma after upfront radiation therapy. In early studies of cyclophosphamide and melphalan followed by AuSCR, the therapy was tolerable in 8 patients with recurrent medulloblastoma with 3 patients experiencing a PR and 1 a CR (ORR 50%).^23^ In another study, 4 of the 19 patients with recurrent medulloblastoma treated with cyclophosphamide and melphalan had no evidence of disease (NED) following AuSCR.^24^ A regimen of high-dose carboplatin, thiotepa, and etoposide followed by AuSCR has also been evaluated; of 23 patients, 7 remained without disease progression at a median of 54 months after AuSCR.^19^ A further report from the expanded series with long-term follow-up noted that event-free survival trended towards improvement in patients who demonstrated chemotherapy-sensitive disease and who received further irradiation as part of salvage therapy.^18^

The German protocol HIT97 enrolled 72 patients with recurrent medulloblastoma and PNET.^25^ Patients received 2 cycles of carboplatin and etoposide followed by disease evaluation. Those who demonstrated response received 2 additional cycles followed by high-dose carboplatin, etoposide, thiotepa and AuSCR, and overall, 52% of patients responded after cycle 2. However, only 5 patients were long-term survivors with 2 patients reported to have a continuous remission. Patients with recurrent medulloblastoma after treatment on HIT-SIOP-PNET4 were treated with combinations of surgery, radiation, and HDC with AuSCR. Five-year OS was 6% and in multivariate analysis neither irradiation nor HDC with AuSCR significantly prolonged survival.^26^

Given the poor prognosis of recurrent medulloblastoma, there remains a need for improved therapies and effective re-induction strategies. In this study, we describe a conventional chemotherapy regimen that is well-tolerated, effective at inducing disease response, and can be successfully used as a precursor to other potential salvage therapies.

## Materials and Methods

We developed a novel re-induction chemotherapy regimen of intravenous irinotecan and cyclophosphamide with oral temozolomide and etoposide. (Table 1). Agents were primarily chosen based on lack of previous exposure. Cyclophosphamide was included for its known efficacy against medulloblastoma and to aid with peripheral blood stem cell (pBSC) collection.

**Table 1.** Re-induction Regimen (Each Cycle is 28 Days)

Drug	Route	Dose	Frequency
Irinotecan	IV	125 mg/m^2^/dose	Days 1, 8
Cyclophosphamide	IV	1500 mg/m^2^/dose	Days 1, 2
Temozolomide	PO	150 mg/m^2^/dose	Qday on days 1–5
Etoposide	PO	50 mg/m^2^/dose	Qday on days 1–7

The primary objectives were analysis of toxicity and ORR; overall survival was evaluated as a secondary objective. Treatment-associated toxicities were assessed and graded as per the National Cancer Institute Common Terminology Criteria for Adverse Events Version 5. Radiographic responses were evaluated by a pediatric neuroradiologist at each institution, as defined by the published Response Assessment in Pediatric Neuro-Oncology criteria: CR with disappearance of all disease, PR with ≥50% reduction in disease, progressive disease (PD) with ≥25% increase in disease, or stable disease (SD) with disease not meeting the aforementioned criteria.^27^

Patients underwent disease evaluation with magnetic resonance imaging after 2 cycles of chemotherapy with the expectation that patients would either receive 2 additional cycles of therapy for PR or proceed to consolidation in the setting of NED. Radiographic disease evaluation was repeated after 4 cycles. For patients treated with HDC and AuSCR upfront, the intent was to give CSI as consolidation. For patients initially treated with CSI, the intent was to give HDC and AuSCR as consolidation therapy. If HDC and AuSCR were planned, pBSC collection was performed after cycle 1, if not previously collected as part of upfront therapy and available for administration.

Approval was obtained from the Children’s Hospital Los Angeles and the University of Alabama at Birmingham institutional review boards (CHLA-21-00219, UAB IRB-300008049) to conduct a retrospective chart review of children with recurrent medulloblastoma treated with the re-induction regimen as described from 2015 to 2020. Clinicopathological features at diagnosis and recurrence as well as toxicity, treatment response, and survival data were collected.

## Results

### Demographics

Nine children were included in this study. Demographics, clinicopathologic features, and initial treatments are outlined in Table 2. The median patient age at diagnosis was 5.75 years (range: 2.5–17 years) and there was a male preponderance (67%). At the time of diagnosis, four patients (44%) had M0 disease per modified Chang staging system, three patients (33%) had M3 disease, and one patient (11%) had M4 disease; one patient had unknown upfront M staging.^4^ Molecular subgrouping was available in 6 patients, and all were non-SHH/non-WNT.

**Table 2.** Demographics and Initial Treatment

Patient	Age at Dx (months)	Sex	Stage	Extent of initial resection	Histology	Group	Upfront radiation	Upfront chemotherapy
1	75	F	M0	GTR	Classic	4	CSI 2340cGy + boost 3060cGy	Maintenance: CCNU, VCR, CP, CPM (9 cycles)
2	53	F	M0	GTR	Classic	3	n/a	Induction: CP, CPM, ETOP, VCR, MTX (6 cycles) Consolidation: HDC carboplatin, thiotepa, etoposide with AuSCR (1 cycle)
3	65	M	M3	STR	Anaplastic	4	Spinal 2310cGy then cranial 1800cGy + boost 5400cGy	Induction: CP, CPM, ETOP, VCR, MTX (3 cycles), metronomic cyclophosphamide and etoposide, maintenance with cis-retinoic acid and vorinostat
4	107	M	M3	GTR	Classic	4	CSI 3600cGy + boost 1980cGy	Maintenance: CP, CPM, VCR (6 cycles)
5	210	M	unk	unk	unk	unk	CSI 4140cGy	n/a
6	119	M	M0	STR	Classic	4	CSI 2340cGy + boost 3060cGy	Maintenance: CCNU, VCR, CP, CPM (9 cycles)
7	31	M	M0	GTR	Classic	3/4	n/a	Induction: CP, CPM, ETOP, VCR, MTX (6 cycles) Consolidation: HDC carboplatin, thiotepa, etoposide with AuSCR (1 cycle)
8	69	F	M3	GTR	Classic	3/4	n/a	Induction: CP, VCR, ETOP, CPM, MTX (3 cycles)
9	36	M	M4	GTR	Anaplastic	Unk	n/a	Induction: CP, VCR, ETOP, CPM, MTX (5 cycles)

M, male; F, female; GTR, gross total resection; STR, subtotal resection; unk, unknown; VCR, vincristine; CP, cisplatin; CPM, cyclophosphamide; ETOP, etoposide; MTX, methotrexate.

### UpFront Therapy

Initial treatment varied among subjects. Four patients were treated with HDC and AuSCR (patients 2, 7, 8, and 9). One patient had a complex induction course due to neurologic compromise and received modified induction chemotherapy in addition to spinal radiation with palliative intent. This was followed by remarkable clinical improvement; therefore, this patient subsequently received whole brain radiation with boost (patient 3). Three patients were treated with upfront CSI and maintenance chemotherapy (patients 1, 4, and 6) while one patient who received initial therapy abroad was treated with CSI alone (patient 5).

### Recurrence and Re-induction

**Figure 1.**
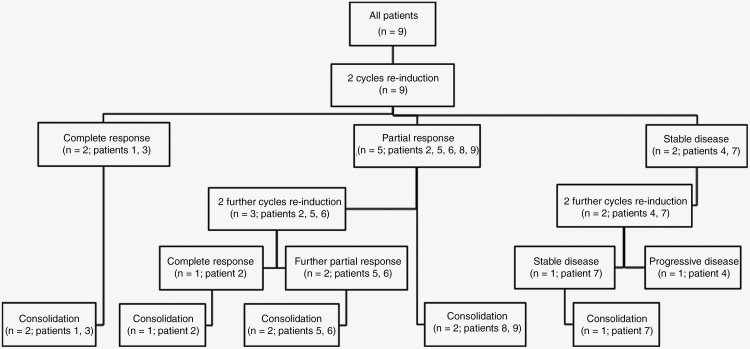
CONSORT diagram.

The mean time from diagnosis to first recurrence was 19 months with 2 patients having both local and leptomeningeal recurrences and 7 patients experiencing distant recurrences at metastatic sites outside the tumor bed. Recurrence and re-induction treatment are outlined in Table 3. All patients received at least 2 cycles of the described re-induction chemotherapy regimen, as outlined in Figure 1.

**Table 3.** Recurrence and Re-induction Treatment

Patient	Time to relapse (months)	Relapse site (LM+/− local)	Total re-induction cycles	Response after 2 cycles (SD/PR/CR/PD)	Response after 4 cycles (SD/PR/CR/PD)	Re-induction toxicities group 3/4 per CTCAE v.5	Consolidation	Time to further progression (months)	Site of recurrence local vs LM	Most recent status
1	23	LM	2	CR	n/a	Febrile neutropenia	HDC carboplatin, thiotepa, etoposide + AuSCR	12	LM	Alive on metronomic antiangiogenic therapy
2	17	LM	4	PR	CR	Febrile neutropenia Sepsis	Cranial 2340cGy + spinal 3600cGy	n/a	n/a	Alive and NED at 56 months
3	29	LM	2	CR	n/a	Catheter-related infection	HDC carboplatin, thiotepa, etoposide + AuSCR Cranial 2340cGy + boost 4500cGy	n/a	n/a	Alive and NED at 80 months
4	20	LM	4	SD	PD	Febrile neutropenia	Not given due to progression	5	LM	Alive on metronomic antiangiogenic therapy
5	15	LM	4	PR	PR	Febrile neutropenia	HDC carboplatin, thiotepa + AuSCR, CSI 2340cGy + boost 3000cGy	n/a	n/a	Died of XRT toxicity, NED at 28 months
6	30	LM	4	PR	PR	Febrile neutropenia Sepsis	HDC carboplatin, thiotepa, etoposide + AuSCR	n/a	n/a	Alive and NED at 15 months
7	16	LM	4	SD	SD	Febrile neutropenia	CSI 3600cGy + boost 1800cGy	11	LM	Metronomic antiangiogenic therapy, maintenance irinotecan bevacizumab now alive and NED at 28 months
8	12	LM + local	2	PR	n/a	Catheter-related infection	CSI 3960cGy + boost 1080cGy	7	LM	Died of disease
9	6	LM + local	2	PR	n/a	Hyponatremia, seizures	CSI 3600cGy + boost 1440cGy	2.5	LM + local	Died of disease

Relapse site is described as local or leptomeningeal (LM). Responses were defined radiographically per RAPNO criteria. CR, complete response; PR, partial response; SD, stable disease; PD, progressive disease. Time to further progression is defined from time of first recurrence. NED, no evidence of disease.

### Toxicities

All patients experienced myelosuppression during re-induction. Additional toxicities during re-induction are described in Table 3. Second to myelosuppression, infection was the most common complication with 6 patients experiencing febrile neutropenia, 2 of whom experienced septic shock requiring admission to the intensive care unit. All patients received therapy at full dose, except patient 4 who required dose modification for persistent thrombocytopenia. There were no treatment-related deaths.

### Response

**Figure 2.**
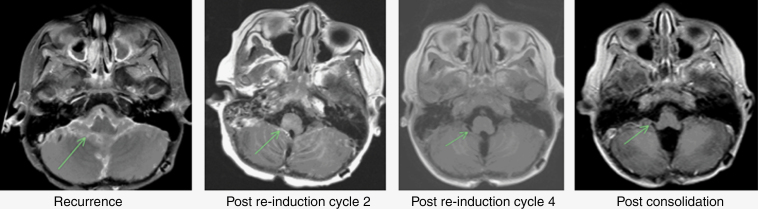
Representative MRI images from patient 2.

**Figure 3.**
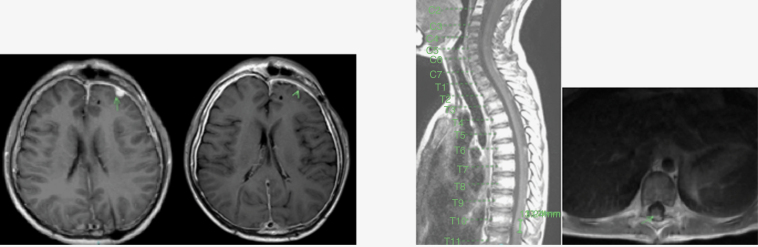
Representative MRI images from patients 5 and 6. Patient 5 (left): Brain lesion prior to re-induction (left) and nearly resolved after 4 cycles (right). Patient 6 (right): T10 lesion prior to re-induction (left) and nearly resolved “plaque” after 4 cycles (right).

Two patients had a CR after two cycles of therapy and proceeded to consolidation (patients 1 and 3). Of the remaining 7 patients, 5 had a PR (patients 2, 5, 6, 8, and 9) and 2 had SD (patients 4 and 7). ORR after 2 cycles was 78%. Two of the 5 patients with PR proceeded directly to consolidation (patients 8 and 9). The other 3 patients who achieved a PR received 2 additional cycles of therapy with 1 patient achieving CR (patient 2, Figure 2) and the other 2 achieving further response resulting in minimal disease (brain lesion nearly resolved in patient 5, spine leptomeningeal plaque nearly resolved in patient 6; Figure 3). The 2 patients with SD received an additional 2 cycles (patients 4 and 7). One patient had ongoing SD after 4 cycles and proceeded to consolidation (patient 7), while the other had PD after cycle 4 (patient 4). In addition, pBSC collection was achieved in all patients for whom it was attempted (*n* = 5).

### Consolidation

All patients were able to reach consolidation, which varied among subjects. The 4 patients initially treated with HDC and AuSCR all received CSI (patients 2, 7, 8, and 9) while the 2 patients initially treated with CSI and maintenance chemotherapy went on to receive HDC and AuSCR (patients 1 and 6). The patient who was treated with induction chemotherapy, palliative spinal, and subsequent low-dose cranial irradiation received HDC and AuSCR followed by whole brain irradiation (patient 3). The patient initially treated with CSI alone proceeded to receive HDC and AuSCR followed by CSI (patient 5). The patient with PD after 4 cycles of re-induction chemotherapy received consolidation as per the MEMMAT protocol (patient 4).^28^

All patients who received consolidation with HDC and AuSCR stem cell rescue had subsequent successful engraftment.

### Survival

Three patients are alive with NED at 15-, 56-, and 80-month follow-up (patients 2, 3, 6). One patient died of consolidation-related toxicity (radiation-induced spinal cord necrosis) but was NED at the time of death 28 months from initial recurrence (patient 5). Five patients eventually developed PD (patients 1, 4, 7, 8, 9). Two patients died of disease, 2 are alive with disease and one is alive with NED after prior PD and additional antiangiogenic plus intrathecal therapy. In total, 4 patients (44%) are alive with NED (patients 2, 3, 6, and 7). Of note, molecular testing was not performed on patients 2 and 3; however, neither showed evidence of *MYC* amplification/overexpression by fluorescence in situ hybridization or immunohistochemistry, respectively. Chromosomal microarray demonstrated that patient 6 had isodicentric 17q and loss of Y chromosome, and patient 7 had copy number gains in chromosomes 1–7, 9, 12–15, and 17–22, with no evidence of *MYC* nor *MYCN* amplification nor isochromosome 17q.

## Discussion

Medulloblastoma requires the use of multimodal therapy in the upfront setting, whether it is surgery in combination with chemotherapy and CSI in older children or surgery in conjunction with conventional chemotherapy and HDC/AuSCR in infants and young children.^29,30^ At the time of relapse, these principles remain true, especially for tumors that have evaded prior intensive therapy. The prognosis at relapse remains very poor and improved therapeutic strategies are needed. Since therapies may be most effective when there is minimal residual disease,^31,32^ it remains important to identify tolerable and effective regimens that may re-induce remission prior to consideration of additional consolidation or clinical trial options.

There are several clinical trials currently underway for recurrent medulloblastoma. A phase II study evaluating metronomic antiangiogenic chemotherapy in the setting of recurrent medulloblastoma is ongoing (NCT01356290) and interim reporting showed a response rate of 45% with 6 patients achieving CR.^33^ The Pediatric Brain Tumor Consortium study PBTC-053 (NCT03904862) is enrolling patients with recurrent SHH group medulloblastoma to evaluate study drug CX-4945, a small molecule and casein kinase 2 inhibitor. The Pediatric Neuro-Oncology Consortium study PNOC027 (NCT05057702) is enrolling patients with recurrent medulloblastoma utilizing sequencing and real-time in vitro drug screening to recommend personalized treatment plans. Biomarker-specific CAR T cell studies for which patients may also be eligible include the IL13Rα2-CAR T Cell trial (NCT04661384) and the BrainChild B7-H3 CAR T trials (NCT04185038 and NCT05768880).

Despite numerous ongoing trials, recurrent medulloblastoma remains challenging to treat. Approaches to minimize disease burden prior to consideration of additional salvage options are needed, and a re-induction regimen as we have outlined can be considered in combination with novel treatment methods that work best in the setting of no or minimal disease.^34–36^

Direct comparisons of response rates are challenging due to the lack of accepted standard of care for recurrent medulloblastoma, and the consequent heterogeneity of treatment approaches. However, most salvage regimens have reported responses of up to ~50%, with temozolomide/irinotecan/bevacizumab reported with an ORR of 47%, HDC approaches including cyclophosphamide/melphalan an ORR of 50%, and HIT97 an ORR of 52%.^14–18,20–26^

In this study we report an ORR of 78%, demonstrating that some patients with recurrent medulloblastoma remain responsive to conventional chemotherapy. Three patients were able to achieve a CR, and two additional patients achieved a substantial PR.

While this investigation was focused on the re-induction chemotherapy regimen, it is worth noting that some patients who received CSI post-re-induction were treated with less than 3600cGy. Patient 2 received 2340cGy cranial and 3600cGy spinal irradiation and is alive with NED at 56 months, while patient 3 received 1800cGy cranial and 2310cGy spinal irradiation followed by 2340cGy cranial irradiation at the time of relapse and is also alive with NED at 80 months. While this is a very small sample size, it is still an important observation given patients may still be young at the time of recurrence and full dose CSI may not align with a family’s goals of care. Given the heterogeneity of the clinical and pathologic features of children with recurrent medulloblastoma, it is important to have a variety of well-tolerated and effective treatment options to offer families based on their goals of care, availability of clinical trial options, and/or other therapeutic modalities.

### Limitations

This study has several limitations including small sample size, heterogeneity of upfront therapy, and incomplete data regarding molecular subgroups which may have influenced response to chemotherapy. It is therefore important to recognize that these results may not be generalizable to all patients with relapsed or progressive medulloblastoma.

### Clinical Application

At our institutions, this regimen is considered in cases of recurrent medulloblastoma—when clinical status allows and in shared decision-making with families—as a bridge to further therapy with curative intent. Potential advantages include induction of response, allowing for further consolidation or consideration of novel therapies and trials with minimal disease. Limitations of this regimen include side effects related to treatment intensity, namely myelosuppression, and the potential for serious infections, which must be considered in the context of a patient’s prior treatment and associated toxicities.

## Conclusion

The described re-induction regimen is well-tolerated and can induce complete and partial responses and lead to subsequent long-term survival when used in combination with consolidation strategies. This regimen should be considered when there is a need to reduce the disease burden prior to proceeding to subsequent treatments that require minimal or no evidence of disease for best outcomes. Further evaluation of this re-induction regimen in a prospective trial is recommended.

## Data Availability

There are no additional data, all data have been presented in the manuscript.
